# Damage Detection in Steel–Concrete Composite Structures by Impact Hammer Modal Testing and Experimental Validation

**DOI:** 10.3390/s22103874

**Published:** 2022-05-20

**Authors:** Viviana Meruane, Sergio J. Yanez, Leonel Quinteros, Erick I. Saavedra Flores

**Affiliations:** 1 Department of Mechanical Engineering, Universidad de Chile, Beauchef 851, Santiago 8370456, Chile; vmeruane@uchile.cl (V.M.); leonel.quinteros@ug.uchile.cl (L.Q.); 2 Civil Engineering Department, University of Santiago of Chile, Av. Víctor Jara 3659, Santiago 8990000, Chile; erick.saavedra@usach.cl; 3 Department of Civil and Environmental Engineering, Imperial College London, London SW7 2AZ, UK

**Keywords:** damage detection, modal testing, composite structure

## Abstract

Steel–concrete composite systems are an efficient alternative to mid- and high-rise building structures because of their high strength-to-weight ratio when compared to traditional concrete or steel constructive systems. Nevertheless, composite structural systems are susceptible to damage due to, for example, deficient construction processes, errors in design and detailing, steel corrosion, and the drying shrinkage of concrete. As a consequence, the overall strength of the structure may be significantly decreased. In view of the relevance of this subject, the present paper addresses the damage detection problem in a steel–concrete composite structure with an impact-hammer-based modal testing procedure. The mathematical formulation adopted in this work allows for the identification of regions where stiffness varies with respect to an initial virgin state without the need for theoretical models of the undamaged structure (such as finite element models). Since mode shape curvatures change due to the loss of stiffness at the presence of cracks, a change in curvature was adopted as a criterion to quantify stiffness reduction. A stiffness variability index based on two-dimensional mode shape curvatures is generated for several points on the structure, resulting in a damage distribution pattern. Our numerical predictions were compared with experimentally measured data in a full-scale steel–concrete composite beam subjected to bending and were successfully validated. The present damage detection strategy provides further insight into the failure mechanisms of steel–concrete composite structures, and promotes the future development of safer and more reliable infrastructures.

## 1. Introduction

Composite structural systems offer a competitive solution when compared to typical concrete or steel constructive systems. Composite system advantages include a higher strength-to-weight ratio, reduced cost due to savings in smaller foundation concrete volumes and thereby reduced earth-moving costs, and better project timing [1,2]. This structural solution has gained popularity in the construction sector as an efficient alternative to mid- and high-rise building floors [3,4,5].

Composite systems are defined as a group of two or more materials that, when used together, lead to improved overall mechanical properties. One of the most common composite structures is a concrete slab attached to an I-section steel beam. The system is constructed by pouring concrete over a steel deck sheet with shear stud anchors welded onto the valleys of the deck, forming a single structural element once concrete is cured [6,7]. The resultant composite system acts as a single structural element, assuming that the shear connection develops its maximal stresses along the length of the beam. However, as concrete is cured, and the load is applied, damage may occur in the material as microcracks or cracks that lead to stiffness deterioration. These phenomena occur in the surrounding area of the connector [8,9,10].

When structural damage occurs in a specific material, its physical properties, such as mass, stiffness and damping, are directly affected. These in turn affect the modal characteristics of structures, natural frequencies, vibration modes, and modal damping [11,12,13]. This relationship allowed for the development of a wide variety of vibration-based structural damage detection techniques [14]. These nondestructive techniques allow for carrying out on-site experimentation, detecting and locating nonvisible damage, and quantifying the degree of damage in a structure [15,16]. In this context, machine learning and deep learning algorithms have been implemented for making an accurate decision on the current structural status of civil engineering systems [17].

These methods are implemented by finding the vibration characteristics by using sensors. Frequency and vibration modes are used as input for algorithms that allow for the identification of damage by comparison. Depending on the type of the used algorithm, it is possible to obtain information about damage, its location in the material, and its severity [16]. Vibration-based damage detection methods need a sample of damaged material and a computational model (traditionally, a finite element model). The method performs analysis on the basis of correlation between experimental data and numerical results, which allows for engineers to identify damage. However, the effectiveness of this solution depends on an accurate representation of the structural response through the computational model, which is normally affected by oversimplifications in the material modeling process, and limited by the capacity of available computational resources. As a consequence, the process might induce errors in the detection of damage when considering the modal data based on linear response of the structure.

Different works have been developed using different vibration characteristics, focusing on vibration mode parameters that are sensitive to structural changes. These methods focus on studying the curvature of vibration modes. Curvature is the second derivative with respect to the position of the modes; therefore, it is an effective way of detecting changes in the physical properties of materials. Pandey et al. [18] showed its effectiveness in one-dimensional structures, and its method was correctly extended to platelike structures using central difference approximation for computations. Wu and Law [19] implemented a damage localization method based on uniform load surface (ULS) curvature changes applied on two-dimensional plate structures. Later, Wu and Law [20] presented a new sensitivity-based method via measured modal parameters to locate and quantify damage in platelike structures. Results showed that changes in the elemental stiffness parameters due to damage gave the location and magnitude of the damaged plate elements. Wang and Qiao [21] applied two methods to the ULS of analytically obtained cracked and delaminated beams, from which damage location and size were determined. Cornwell et al. [22] showed the behavior for different damage scenarios on plates in two dimensions. However, results exhibited difficulties in the algorithm in detecting multiple damages with different degrees of severity. The damage stage could somestimes not be paired with the pristine state. Researchers proposed gapped smoothing [23] (GS) and wavelet-based [24,25] analysis to solve that problem.

One of the main limitations of model-based methods is the lack of flexibility in setting up experiments, and the difficulty in finding a perfectly intact material. To this end, a vibration-based algorithm not based on models was run here to evaluate the damage in a typical composite steel–concrete beam. The aim of this investigation is to assess the applicability of a curvature-based damage detection method to diagnose damage in steel–concrete composite beams. This type of structure is frequently used as the main load-bearing girder of bridge structures. Damage in these structures is often internal and not detectable by inspections, thus causing security risks during their operation. Results of this study represent a first step towards the development of more accurate nondestructive methods to quantify the performance of a structure after damaging conditions in composite steel–concrete beams. In addition, results are validated by experimental tests.

This paper is organized as follows. The damaged index calculated from mode shape curvatures is presented in Section 2. The experimental procedure, the experimental modal testing, and data acquisition technique are detailed in Section 3. The main results obtained with the present method are discussed and analyzed in Section 4. Lastly, Section 5 summarizes the main conclusions and recommendations of the present work.

## 2. Damage Index from Mode Shape Curvatures

The mode shape curvature method can efficiently predict and detect the location of a damaged region [18,26]. When there is no damage, mode shapes typically show a smooth surface. On the other hand, when there is damage in the structure, stiffness is reduced, resulting in sharp changes in the slope. These changes might appear at the damaged location, and the curvature consequently varies.

Since there is evident change in the mode shape curvature in the vicinity of damage, a change in curvature was adopted to locate damage. For 1D structures, the curvature of the *r*-th mode shape at the *i*-th test point can be obtained using the central difference approximation as follows [18]:

(1) $$\frac{\partial^{2}\phi_{r}\left(x_{i}\right)}{\partial x^{2}}=\frac{\phi_{r}\left(x_{i+1}\right)+\phi_{r}\left(x_{i-1}\right)-2\phi_{r}\left(x_{i}\right)}{h^{2}}$$

where $\phi_{r}\left(x_{i}\right)$ is the *r*-th mode shape, and *h* is the uniform separation of the test points in the central difference method. The damage index is computed as the absolute value of the difference between the curvatures of the damaged and undamaged structures:

(2) $$d_{r}\left(x_{i}\right)=|\frac{\partial^{2}\phi_{r}^{\;D}\left(x_{i}\right)}{\partial x^{2}}-\frac{\partial^{2}\phi_{r}^{\;U}\left(x_{i}\right)}{\partial x^{2}}|$$

in which superscript *D* refers to the damaged stage, and *U* to the undamaged case.

In 2D structures, the curvature at point $\left(x_{i},y_{j}\right)$ along each direction of the perpendicular grid lines of the method is obtained as follows:

(3) $$\frac{\partial^{2}\phi_{r}\left(x_{i},y_{j}\right)}{\partial x^{2}}=\frac{\phi_{r}\left(x_{i+1},y_{j}\right)-2\phi_{r}\left(x_{i},y_{j}\right)+\phi_{r}\left(x_{i-1},y_{j}\right)}{h_{x}^{2}}$$

(4) $$\frac{\partial^{2}\phi_{r}\left(x_{i},y_{j}\right)}{\partial y^{2}}=\frac{\phi_{r}\left(x_{i},y_{j+1}\right)-2\phi_{r}\left(x_{i},y_{j}\right)+\phi_{r}\left(x_{i},y_{j-1}\right)}{h_{y}^{2}}$$

where $h_{x}$ and $h_{y}$ are the uniform spacing along each perpendicular direction defined by the grid lines. The damage index can be expressed as

(5) $$d_{r}\left(x_{i},y_{j}\right)={\left(|\frac{\partial^{2}\phi_{r}^{\;D}\left(x_{i},y_{j}\right)}{\partial x^{2}}-\frac{\partial^{2}\phi_{r}^{\;U}\left(x_{i},y_{j}\right)}{\partial x^{2}}|+|\frac{\partial^{2}\phi_{r}^{\;D}\left(x_{i},y_{j}\right)}{\partial y^{2}}-\frac{\partial^{2}\phi_{r}^{\;U}\left(x_{i},y_{j}\right)}{\partial y^{2}}|\right)}^{2}$$

For *m* vibration modes, the damage index at each test point $\left(x_{i},y_{j}\right)$ is calculated as follows:

(6) $$d\left(x_{i},y_{j}\right)=\sum_{r=1}^{m}d_{r}\left(x_{i},y_{j}\right)$$

An additional index corresponds to the sum of all the damage index and is expressed as

(7) $$I=\sum_{i=1}^{o}\sum_{j=1}^{p}\sum_{r=1}^{m}d_{r}\left(x_{i},y_{j}\right)$$

where *o* and *p* are points of form (*x*,*y*).

### Mode Shape Pairing and Scaling

To compute the damage index in Equation (7), it is necessary to match the damaged modes with corresponding undamaged ones by calculating the modal correlation given by the modal assurance criterion (MAC), defined as:

(8) $$MAC_{ij}=\frac{{\left(\phi_{iU}^{T}\phi_{jD}\right)}^{2}}{\left(\phi_{iU}^{T}\phi_{iU}\right)\left(\phi_{jD}^{T}\phi_{jD}\right)}$$

where $\phi_{iU}$ is the *i*-th undamaged mode, $\phi_{jD}$ is the *j*-th damaged mode, and the superscript *T* denotes the vector transpose. This correlation provides a number ranging from 0 to 1, where 0 is null correlation and 1 is a perfect correlation. The advantage is that it depends on the shape of the mode and not on the scale. Therefore, a minimal MAC value can be set to affirm that two modes in a set of undamaged and damaged modes correspond to the same modal shape.

After pairing, mode shape pairs must be consistently normalized with the modal scale factor (MSF) [27,28]. The MSF measures the scale factor between two modes; thus, damaged modes can be scaled to undamaged ones by multiplying by the corresponding MSF using the following expressions:

(9) $$\phi_{jD}^{*}=\phi_{jD}\;\cdot \;MSF_{ij}$$

(10) $$MSF_{ij}=\frac{\phi_{iU}^{T}\phi_{jD}}{\phi_{jD}^{T}\phi_{jD}}$$

where $MSF_{ij}$ is the modal scale factor between the *i*-th undamaged mode and *j*-th damaged mode.

Multiplying the damaged mode by the corresponding MSF solves the problem of the undamaged and damaged modes being with a 180${}^{\circ }$ phase shift.

## 3. Experimental Setup

The experimental framework consisted of a full-scale composite concrete–steel beam designed according to Eurocode 4 [29]. The effect of shear stud anchor local behavior on the overall response was investigated. Data were obtained until the specimen had suffered an evident failure.

### 3.1. Specimen Geometry and Materials

**Figure 1:**
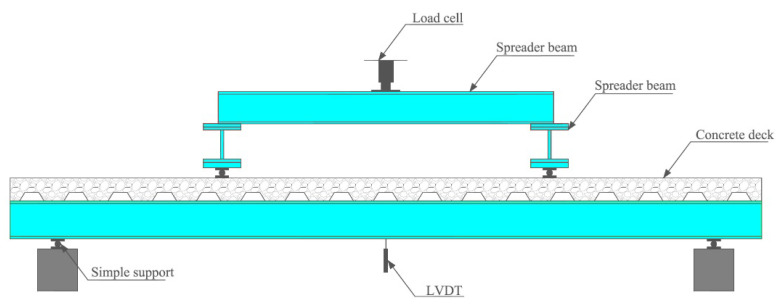
Composite concrete–steel beam test setup.

A schematic representation of the experimental test setup is shown in Figure 1. Concrete slab width was 1300 mm, and length was 5500 mm. A 170 mm thick slab was used for the test. This thickness is common in midrise buildings and parking garages. The specimen was designed following AISC specifications [30], but limited to the steel frame capacity of the structural laboratory. The steel section was simply supported at both ends to simulate the typical end connection of the composite system.

**Figure 2:**
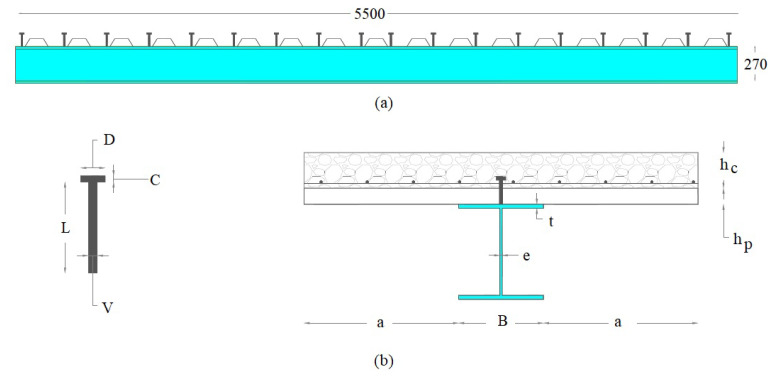
Geometry of composite system: (**a**) length and height of the section; (**b**) beam cross-section and stud anchor. Unit: mm.

An HEA280 steel section was used in the experiment. The steel grade was ASTM A-36 with yielding strength ($\sigma_{y}$) and ultimate strength ($\sigma_{u}$) of 250 and 400 MPa, respectively. The flange width/thickness ratio of the section (b/t=21.5) was smaller than the limit specified for plastic design in the AISC specification [30]. This was justified since the compression flange was longitudinally connected to the concrete slab, and local buckling does not occur. Height, flange width, web thickness, and flange thickness were 270, 280, 8, and 13 mm, respectively (see Figure 2 and Table 1). Total length between supports was of 4800 mm.

The concrete slab was grade G-25 according to American code ACI 211 [31], and its w/c ratio was 0.54. Concrete strength was experimentally obtained from cylindrical molds at 7, 96, and 181 days from casting. The corresponding strength grades were experimentally obtained in cylindrical concrete specimens subjected to pure compression until failure. The experimental procedure is standardized and described in the Chilean national standard NCh170/2016 [32]. Average compressive strength was 29.8 MPa. The concrete slab was connected to the steel section by 18 shear stud anchors of 19 mm in diameter of grade ASTM A-108 (more geometric details are presented in Table 1) to transfer the shear forces produced at the concrete–steel beam section interface. The connection also prevented the slip of the steel to a concrete slab.

A 0.8 mm thickness steel deck profile of grade A 653SQ Gr.37—G. 90 was installed to substitute both the concrete slab reinforcement steel bars and the typical framework system [33]. The steel deck was placed transverse to the beam length as used in typical composite construction. Once the deck had been placed, the area between steel section and decking was greased to help in reducing friction effects.

### 3.2. Testing Procedure

During the experimental procedure, a single-ended ENERPAC actuator, model SCR506H, of 50 ton capacity, was connected to a KYOWA load cell, model C-50TE, of 50 ton capacity. We also used two ENERPAC hydraulic pumps, model ZU4, to control actuator displacement and force.The actuator transferred the loading data to homemade scanner equipment that stored the applied-force and displacement data. Stored data were visualized in a LABVIEW software interface. To obtain the midspan displacement of the composite beam, we placed two microsensor LVDTs, model DC750-2000, into each side of the beam, near the neutral axis. The load was evenly distributed in two spreader steel beams to replicate a typical four-point bending test (see Figure 1). For the four-point bending test, a pure bending condition could be achieved in the beam segment near the midspan.

**Figure 3:**
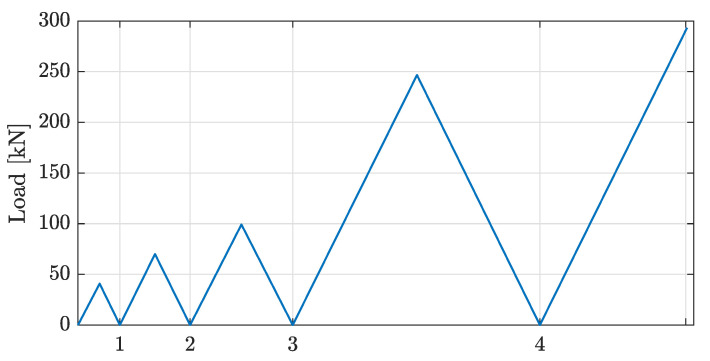
Loading protocol for the experiment.

The loading protocol consisted of four cycles of monotonic increments of 40 kN ( 4 Tonf), 70 kN ( 7 Tonf), 100 kN ( 10 Tonf), and 250 kN ( 25 Tonf). For each increment, the load was completely released until 0 kN had been measured. The subsequent load increment was then imposed until evident failure of the system had been observed (i.e., the load vs. slip curve dropped 10% of the maximal load). The loading protocol is shown in Figure 3, which was taken directly from [34]. However, the actual experiment was interrupted between each loading or unloading cycle in order to measure vibration parameters with the impact hammer modal testing procedure.

A linearly varying displacement transducer (LVDT) supported on an independent support device was placed below the midspan bottom face of the specimen to measure midspan deflection. During the tests, applied load and deflection were simultaneously recorded.

### 3.3. Modal Testing

The methodology used to obtain the frequency response functions (FRFs) consisted of the impact hammer modal test, which is a common technique and consists of having a structure with sensors in measuring points to capture displacement due to an“instant” hit by a hammer in the excitation point. Measuring and excitation point locations are fundamental, their number must be enough to obtain independent modal shapes, and they must be placed in the maximal displacement points to be identifiable.

**Figure 4:**
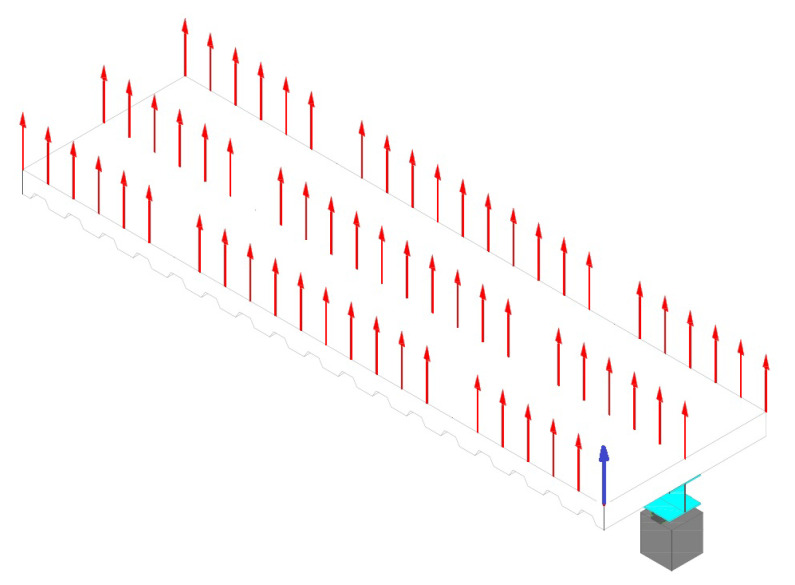
Excited point distribution on steel deck (red arrows). Blue arrow indicates sensor location.

**Figure 5:**
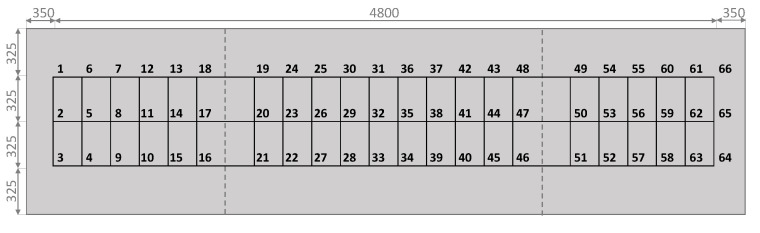
Plan view of slab with numbering of excited points. Unit: mm.

**Figure 6:**
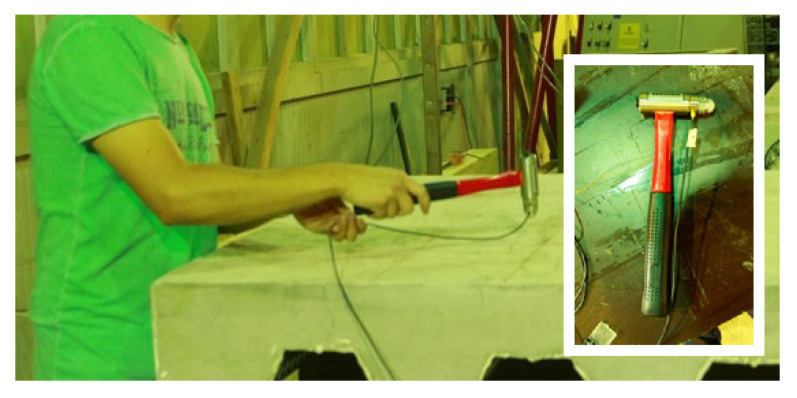
Photograph of the actual experiment in which the hammer modal testing procedure is applied to the concrete-steel beam. The inset figure shows the hammer used to measure vibration properties.

In the experimental set, the composite steel beam was divided into a 3 × 12 grid points as shown in Figure 4. The accelerometer was placed in a corner where large displacements were expected. The excitation and response orientations were parallel to the longitudinal (Z) axis. Figure 5 shows the plan view of the concrete slab with the numbering of the excited points. Figure 6 shows a photograph of the actual experiment in which the hammer modal testing procedure is applied to the concrete-steel beam.

**Figure 7:**
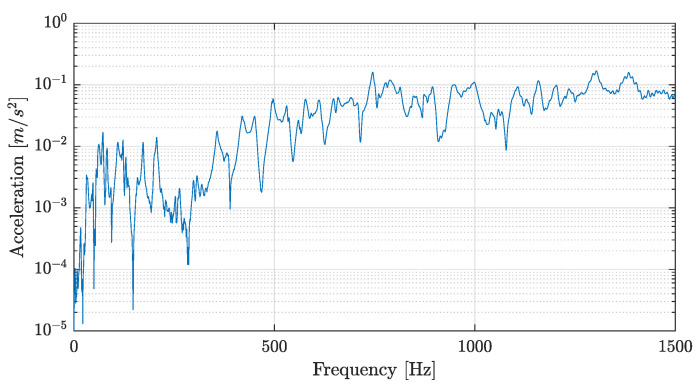
FRF example from the first accelerometer in the initial state.

**Figure 8:**
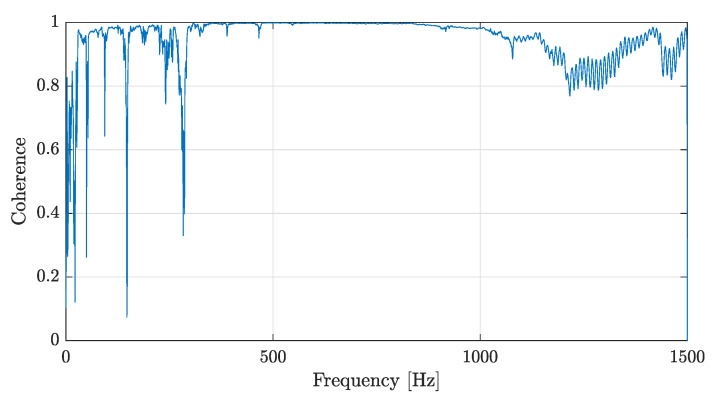
Coherence example from the first accelerometer in the initial state.

First, a frequency range was selected from 1 to 1500 Hz (avoiding rigid body modes) to take the impact test data. Then, a frequency range with coherence close to 1 was selected. Coherence indicates how much of the output is due to the input in the FRF; it is frequently used as an indicator of the quality of the FRF (when it is close to 1). The selected frequency interval was between 100 and 600 Hz. The same frequency range was used for all damage states that included the structure after the following loads had been applied: 40, 70, 100, 150, and 250 kN. An additional experimental lecture of 150 kN was added into the loading protocol to capture the damage between the fourth cycle and failure.Typical FRF data and their corresponding coherence are shown in Figure 7 and Figure 8, respectively.

**Figure 9:**
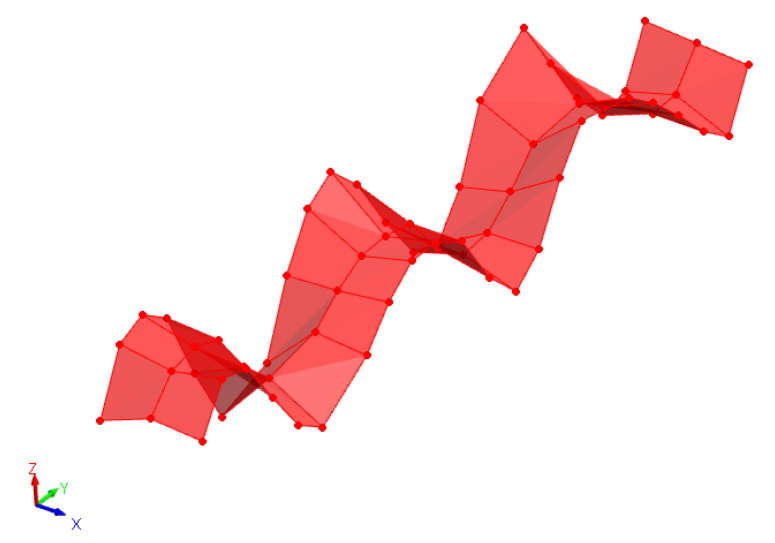
Initial state mode shape ω=278.38Hz.

The measured data (frequency response functions) were imported to commercial software FEMtools 3.8.1 [35], which was able to deal with the relevant dimensions of all the datasets in a reasonable amount of time [36,37,38]. By defining the experimental set as shown in Figure 4, the software could extract the modal parameters from the FRFs using a global polyreference least-squares complex frequency (pLSCF) method or a local curve-fit method. An example of an identified mode shape is shown in Figure 9.

## 4. Results

### 4.1. Load–Displacement Behavior from Static Loading Test

**Figure 10:**
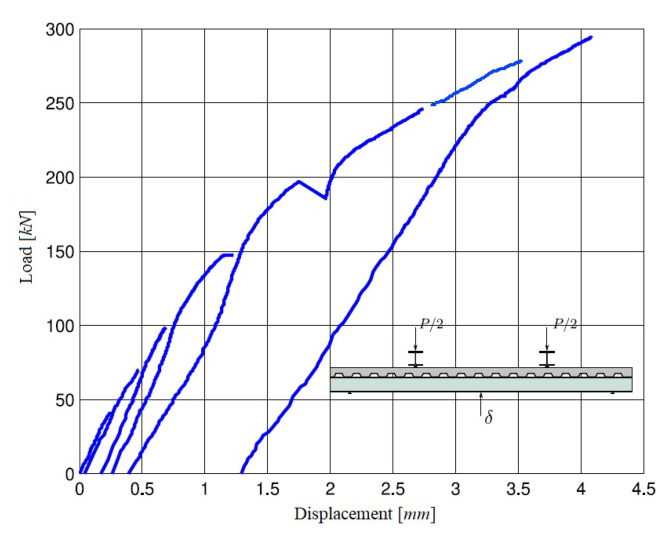
Load–displacement curve for composite steel–concrete beam.

The load–displacement curve of the steel–concrete composite beam is shown in Figure 10, where the abscissa is the displacement monitored from the midspan of the specimen, and the ordinate is the load applied by the testing machine. The vertical displacement of the specimen is equal to the average of the values measured by the LVDTs placed at both sides of the beam midspan.

According to European code EC4 [29], the bending capacity of the structure is 586kNm, and yielding the full cross-sectional area was expected. At that point, a plastic hinge formed at the locations of the maximal bending stress, and eventually the system collapsed. During this experimental procedure, bending capacity was maintained at the lower levels of the nonlinear regime. However, the structure first exhibited linear behavior during the elastic stage at low loading conditions, whereas nonlinear behavior could be observed when the load was increased, and cracking or microcracking in the concrete became visible.

### 4.2. Modal Correlation

**Figure 11:**
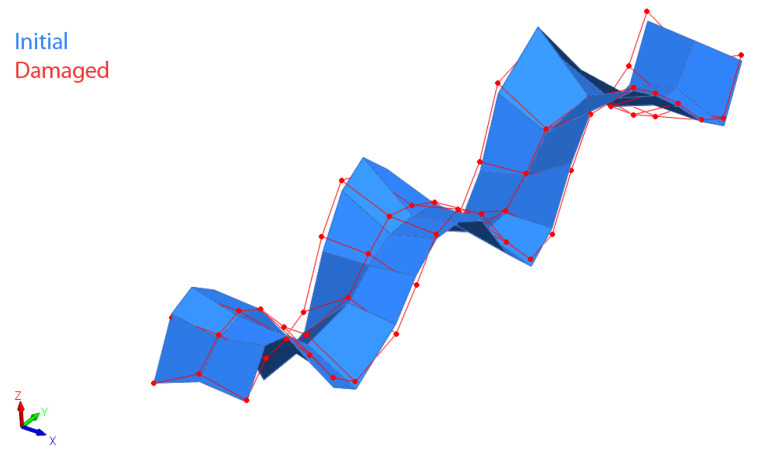
Example of a pair of initial and damaged modes for 40 kN state.

Each mode between damaged and initial states was correlated. This procedure yields a number of correlated pairs for each load case, chosen by MAC values over 80%. Essentially, the damaged mode is correlated to the initial mode when the MAC percentage is greater than 80%. A good modal correlation level was assumed when MAC was at least 80%. Other authors adopted the same criterion [39,40]. The damaged mode does not necessarily coincide with the same initial mode. The mode order varies in the presence of damage because new local modes might appear, in addition to the fact the frequency variation is not the same for all modes; therefore, modes with similar frequency values might switch order after damage is introduced. For example, Table 2 shows the list of correlated mode pairs for a corresponding load of 40 kN. MAC varied from 83.8% for damaged modes 21 and 27, to a MAC percentage of 98.4% for damaged and initial modes 22 and 28, respectively. Table 3 shows the list of correlated mode pairs for a corresponding load of 250 kN. In this case, the MAC varies from 82.4% for damaged modes 12 and 16, to a MAC percentage of 96.8% for damaged and initial modes 26 and 31, respectively. For increasing damage, correlated mode pairs show increasing differences in their frequencies. For a vertical load of 40 kN, only small percentage differences were found, with a maximal percentage difference of 1.71%. However, for a load of 250 kN, the maximal difference increased up to 8.53%. Figure 11 shows an example of a pair of initial and damaged modes for a vertical load of 40 kN.

Only pairs where the initial mode was present in all damaged states were considered. This methodology was adopted to link the changes in the initial modes to all loading cases. Selected pairs are summarized in Table 4, and Figure 11 shows an example of a pair of initial and damaged modes for the 40 kN loading condition.

### 4.3. Damage Intensity

**Figure 12:**
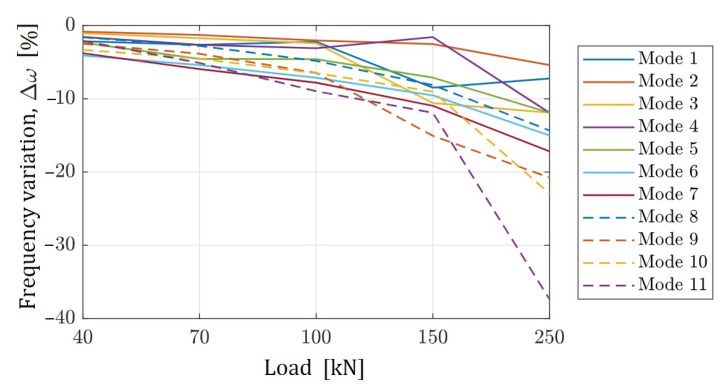
Change in natural frequencies.

The intensity of damage can be described by measuring the reduction in frequency response in each mode due to the reduction in structural stiffness in the cracked zones, or by calculating the damage index defined in Section 2. Figure 12 shows the reduction in natural frequencies for modes 1 to 11. Mode 11 reduced the frequency by almost 40% compared with the undamaged condition. In all other modes, variation in the frequency response varied from 6% to 22% of the initial response.

**Figure 13:**
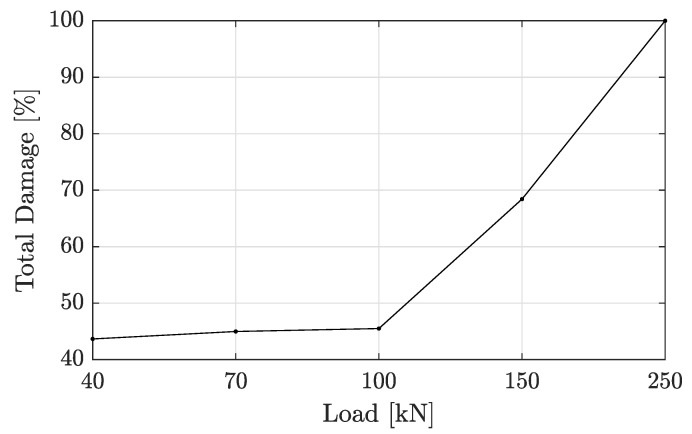
Increase in damage index.

On the other hand, the damage index for each mode was calculated by using Equation (9), and the total damage index for each group was then obtained by using Equation (10). The total damage index is plotted for different loading states, varying from an initial 40 kN to a final load of 250 kN as shown in Figure 13. This figure shows that the more severe the damage level is, the higher the damage index is. This behavior indicates, that if a small amount of damage is not visually detectable, the possibility of failure nonlinearly increases with damage evolution. To this extent, damage in the structure can be estimated from the damage index relationship. However, more exact relationship curves are challenging to obtain and may vary with different beam damage locations. This condition was observed by Zhou et al. [41].

### 4.4. Damage Location

**Figure 14:**
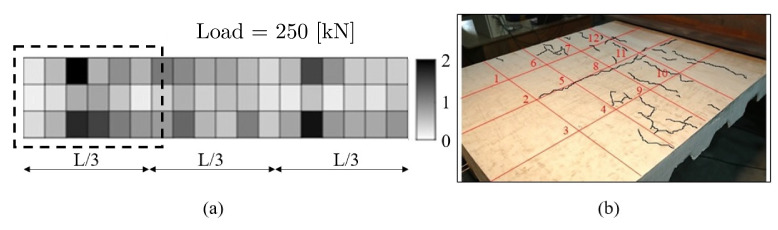
(**a**) Whole beam with damage indices computed with the present numerical approach. (**b**) Experimentally measured crack pattern. (left) Area on the right highlighted.

The damage index at each measured point is visualized in Figure 14. Each zone represents an specific damaged index where a gray scale value was assigned. To represent high values of the damage index, dark shades were used, and low values of the damage index are represented by light shades of gray. It is evident that high-damage indices corresponded to a higher probability of damage in that region. At the onset of the load, the specimen suffered from damage on multiple locations due to the loss of smoothness given by the growth and propagation of cracks. In loading conditions between 40 and 100 kN, the damaged index showed low sensibility, indicating almost constant damage. However, for loading greater than 150 kN, damage substantially incremented, resulting in darker tones evenly spread throughout the structure. Lastly, the specimen was highly damaged when it had been loaded with 250 kN. The damage index reached a higher value in the area surrounding the locations of the load and near the supports.

In Figure 14, the right image shows only one-third of the total length of the beam, which was focused on the crack pattern located on the left support, where the greatest damage was located. The central part of the beam is not shown given the very limited (or nearly zero) damage present there. Both left and right images show good overall agreement between our numerical predictions and the experimentally measured crack pattern, revealing the predictive capabilities of the proposed methodology.

## 5. Conclusions

This paper presented a mathematical framework to localize regions with reduced stiffness in a composite steel–concrete beam by using an impact-hammer-based modal testing procedure. The mathematical formulation adopted in this work allowed for the identification of regions where stiffness varied with respect to an initial virgin state. A stiffness variability index based on two-dimensional mode shape curvatures was generated for several points on the structure to capture the damage distribution pattern.

A correlation procedure for each mode between the damaged and initial states was performed by implementing the modal assurance criterion (MAC) and the modal scale factor (MSF). Since the method relies on adequate mode shape pairing and scaling among undamaged and damaged modes, the procedure compared well with experimentally measured data in a full-scale steel–concrete composite beam. As a consequence, data preprocessing was crucial to compute the damage indices.

The intensity of damage was described by measuring the reduction in the frequency response in each mode due to the reduction in structural stiffness in the cracked zones. A 40% reduction in natural frequencies was observed when compared with the undamaged condition. In general, variation ranged from 6% to 22% of the initial response.

The damage and total damage indices were obtained and plotted for different loading states. The more severe the damage level was, the higher the damage index was. Therefore, damage in the structure was estimated from the damage index relationship.

Numerical damaged-area predictions were successfully compared with experimental data in the composite beam subjected to bending, although numerical studies should be performed in future works to ensure the effectiveness of the framework. The present damage detection strategy can provide further insight into the failure mechanisms of steel–concrete composite structures.

## Figures and Tables

**Table 1:** Geometric parameters of composite beam and shear stud anchor.

Element	Parameter	Value [mm]
Composite beam	$h_{c}$	117.0
	$h_{p}$	53.0
	*e*	8.0
	*a*	510.0
	*B*	280.0
	*t*	13.0
	$h_{a}$	73.0
	*H*	270.0
Stud anchor	*D*	31.8
	*C*	10.0
	*L*	110.0
	*V*	19.0

**Table 2:** List of correlated mode pairs (MAC > 80%), for 40 kN damage load.

Initial Mode ID	Hz	Damage Mode ID	Hz	Diff. (%)	MAC (%)
10	109.11	8	107.24	−1.71	86.9
12	122.57	9	122.94	0.30	83.9
15	155.53	10	153.39	−1.37	87.6
16	171.86	11	161.19	−6.21	91.9
17	198.69	13	197.71	−0.49	94.2
18	206.70	14	205.87	−0.40	95.8
21	253.70	16	253.40	−0.12	95.1
22	263.56	17	262.51	−0.40	96.8
23	279.27	18	278.37	−0.32	91.5
24	297.38	19	295.83	−0.52	93.8
27	323.18	21	321.20	−0.61	83.8
28	356.74	22	354.40	−0.66	98.4
29	385.36	23	381.28	−1.06	97.4
30	419.65	24	415.87	−0.90	97.8
31	451.10	25	449.50	−0.35	98.1
32	494.66	26	492.18	−0.50	97.8
33	531.86	28	528.56	−0.62	97.7
35	560.22	30	557.55	−0.48	93.7
37	576.38	31	574.29	−0.36	98.1

**Table 3:** List of correlated mode pairs (MAC > 80%), for 250 kN damage load.

Initial Mode ID	Hz	Damaged Mode ID	Hz	Diff. (%)	MAC (%)
13	129.42	9	118.38	−8.53	87.7
15	155.53	11	148.30	−4.65	89.4
16	171.86	12	160.06	−6.87	82.4
18	206.70	14	201.31	−2.61	94.4
22	263.56	17	251.68	−4.51	87.0
24	297.38	20	285.48	−4.00	88.0
28	356.74	23	344.84	−3.34	84.6
29	385.36	24	370.36	−3.89	83.5
30	419.65	25	402.47	−4.09	93.0
31	451.10	26	436.78	−3.17	96.8
32	494.66	27	473.91	−4.20	96.0
33	531.86	28	508.94	−4.31	93.2
37	576.38	29	539.03	−6.48	87.5

**Table 4:** Pair selection-criteria summary.

Initial State			40 kN Load			70 kN Load			100 kN Load			150 kN Load			250 kN Load	
Mode ID	Freq. [Hz]		Mode ID	Freq. [Hz]		Mode ID	Freq. [Hz]		Mode ID	Freq. [Hz]		Mode ID	Freq. [Hz]		Mode ID	Freq. [Hz]
15	155.5		10	153.4		12	152.9		11	159.7		12	147.1		11	148.3
16	171.8		11	161.2		13	159.5		12	164.7		13	159.3		12	160.1
18	206.7		14	205.9		16	205.4		14	204.7		15	204.2		14	201.3
22	263.5		17	262.5		19	261.8		16	261.1		16	253.0		17	251.7
24	297.3		19	295.8		21	294.8		18	294.3		19	295.8		20	285.5
28	356.7		22	354.4		24	352.2		22	352.2		22	349.6		23	344.8
29	385.3		23	381.3		26	380.0		23	378.3		23	375.8		24	370.4
30	419.6		24	415.9		27	413.7		24	411.9		24	408.7		25	402.5
31	451.1		25	449.5		28	448.3		25	446.3		25	443.0		26	436.8
32	494.6		26	492.2		29	490.8		27	488.2		26	479.6		27	473.9
33	531.8		28	528.6		31	527.3		28	525.4		28	522.9		28	508.9
37	576.3		31	574.3		34	571.3		31	567.4		30	564.5		29	539.0

## Data Availability

Not applicable.
